# Supplementary material for: Tissue-intrinsic beta-catenin signals antagonize Nodal-driven anterior visceral endoderm differentiation
Source: Nat Commun. 2024 Jun 13;15:5055. doi: 10.1038/s41467-024-49380-0 (PMC11176336; doi:10.1038/s41467-024-49380-0)
Supplement: Supplementary file 1 — Supplementary Information [file 41467_2024_49380_MOESM1_ESM.pdf]

Tissue-intrinsic beta-catenin signals antagonize Nodal-driven anterior visceral endoderm differentiation

Sina Schumacher, Max Fernkorn, Michelle Marten, Rui Chen, Yung Su Kim, Ivan Bedzhov, and Christian Schröter

Supplementary Information

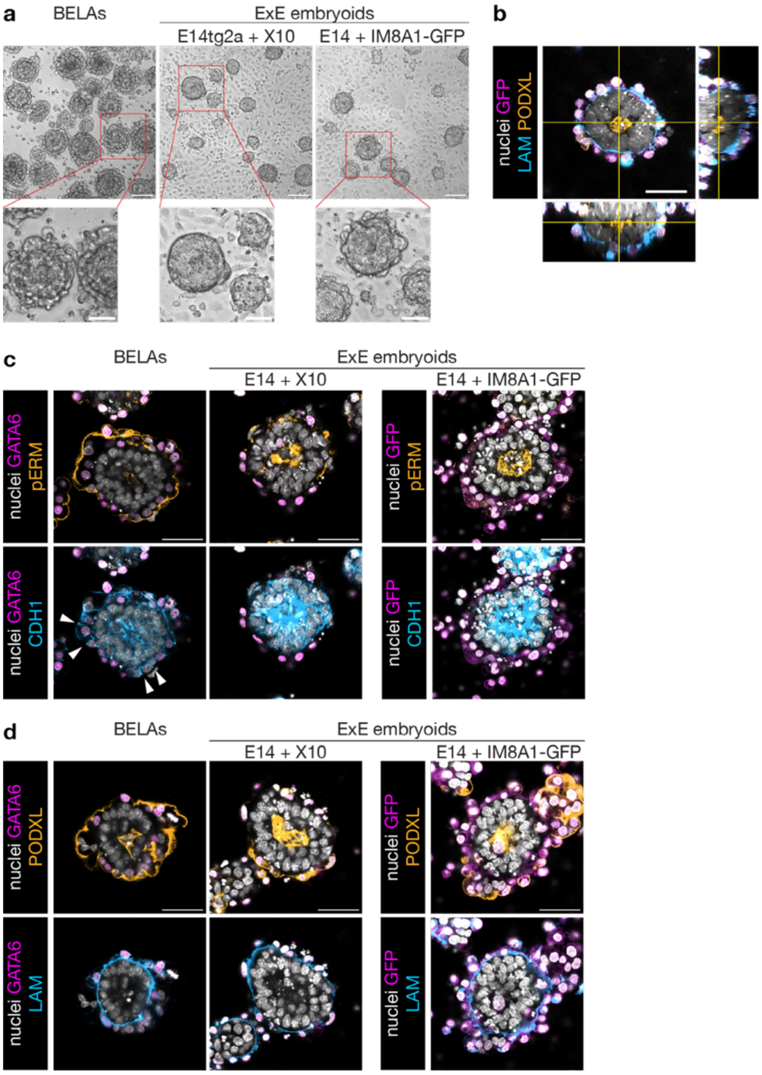

Supplementary Fig. 1. Comparison of BELAs and EXE embryoids, related to Fig. 1.
a Transmitted light images of BELAs (left) and EXE embryoids[12] (middle and right) formed by co-culture of ESCs and XEN cells on gelatin-coated plastic in N2B27. One representative out of n = 2 independent experiments shown. **b** Orthogonal views of an ExE embryoid generated from E14 ESC and IMA8A1-GFP XEN cells, and immunostained for GFP to mark XEN cells (magenta), the basement membrane marker LAM (blue), and the apical marker PODXL (orange). One representative of n = 3 independent structures shown. **c**, **d** Immunostainings of BELAs and EXE embryoids for the PrE/VE marker GATA6 (magenta), the apical markers pERM (**b**) and PODXL (**c**) (orange), the epithelial marker CDH1 (**b**), and the basement membrane marker LAM (**c**) (blue). For EXE embryoids generated with IM8A1-GFP XEN cells, GFP is stained instead of GATA6 to mark XEN cells. Nuclei shown in gray in (**b** - **d**). Arrowheads in (**c**) indicate CDH1 expression in GATA6-positive VE cells in BELAs. One representative out of at least n = 10 structures shown. Scale bars: 100 μm ((**a**), overview); 50 μm ((**a**) insets, (**b** - **d**)).

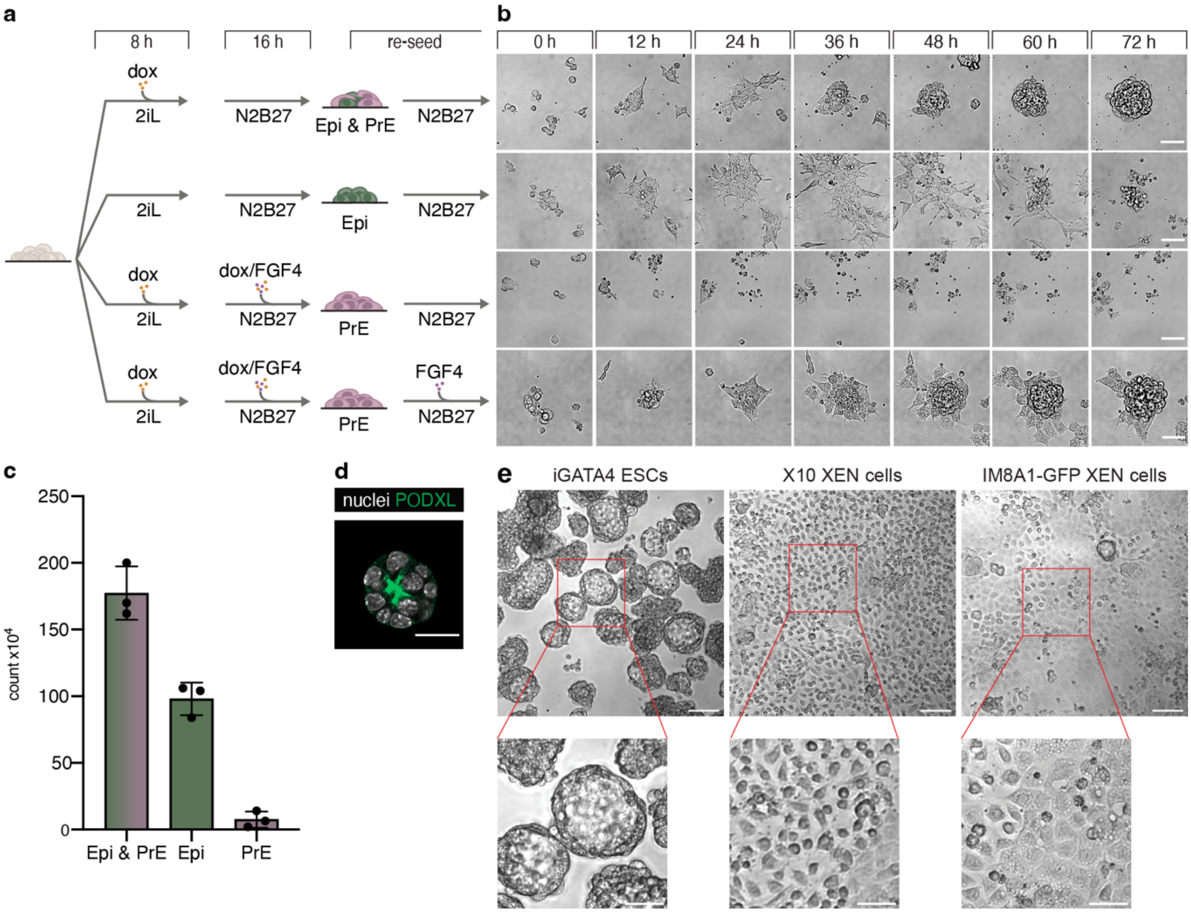

Supplementary Fig. 2. Differentiation of pure populations of Epi and PrE cells, related to Fig. 1. **a** Experimental approach to differentiate pure populations of Epi and PrE cells. Top row indicates method to generate mixed cultures of Epi and PrE cells for comparison. **b** Stills from movies of the cell types differentiated as in (**a**) after re-seeding in N2B27. One representative out of n = 3 independent experiments shown. **c** Quantification of live cells differentiated as in (**a**) without addition of exogenous FGF4 (first three conditions in (**a**)) three days after re-seeding. n = 3 independent experiments. **d** Immunostaining for the polarization marker PODXL in mESCs seeded in matrigel and cultured for 2 days in N2B27. One representative out of n = 20 independent structures shown. **e** Transmitted light images of VE cysts formed from pure populations of PrE cells differentiated from ESCs (left), and monolayers of two different XEN cell lines cultured under the same conditions. One representative out of n = 2 independent experiments shown. Scale bars: 25 µm ((**b**), (**d**)); 100 µm ((**e**), overview); and 50 µm ((**e**), insets). Source data for (**c**) are provided in the Source Data file.

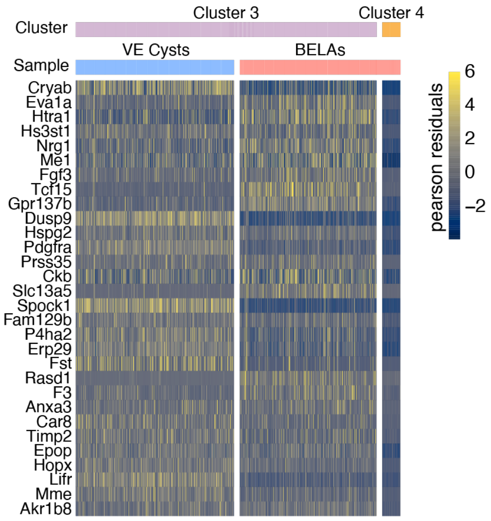

Supplementary Fig. 3. Down-regulated genes in cluster 4 from Fig. 3a.
Heatmap showing the 30 most down-regulated genes between the cells of cluster 3 and cluster 4 from Fig. 3a, ordered by log2-fold change. Single-cell expression is shown as the Pearson residual of the normalized counts. Source data are provided as Supplementary Data 1.

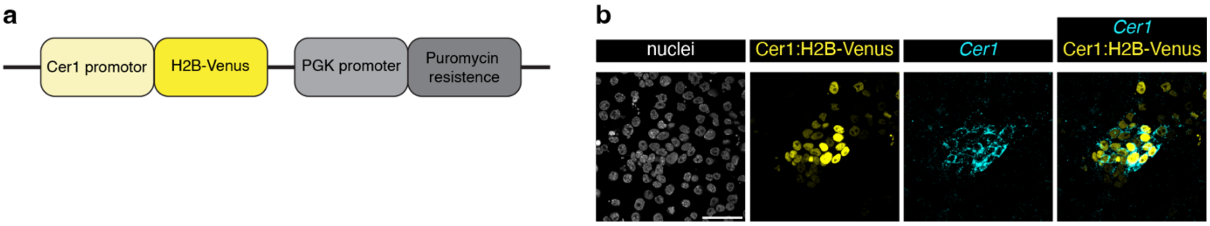

Supplementary Fig. 4. Design and validation of a Cer1:H2B-Venus reporter construct, related to Fig. 3.

a Schematic of the Cer1:H2B-Venus reporter construct. A 4-kb regulatory region of the *Cer1* gene[1] was fused to an H2B-Venus reporter, coupled to a puromycin resistance cassette, and integrated into inducible cells via piggybac transgenesis. **b** Co-expression of H2B-Venus protein (yellow) and Cer1 mRNA (cyan) stained by in situ HCR. Shown is a single confocal section of Cer1:H2B-reporter cells differentiated towards PrE and treated with 50 ng/ml Activin A after removal of 2i medium. Nuclei are labeled with Hoechst33342. Scale bar: 50 µm.

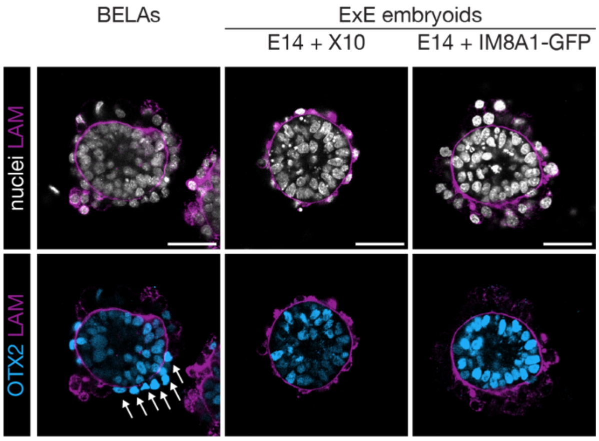

Supplementary Fig. 5. AVE differentiation is specific to BELAs, related to Fig. 3.
Immunostaining of BELAs (left) and EXE embryoids made with X10 (middle) or IM8A1-GFP XEN cells (right) for OTX2 (blue) and LAM (magenta). Arrows highlight AVE cells in BELAs based on their OTX2 expression and position outside the laminin ring. Number of structures with OTX2-positive cells outside the laminin ring: 11/12 for BELAs, and 0/10 for both types of EXE embryoids. One representative out of a total of at least 20 structures from n = 2 independent experiments shown. Scale bars: 50 μm.

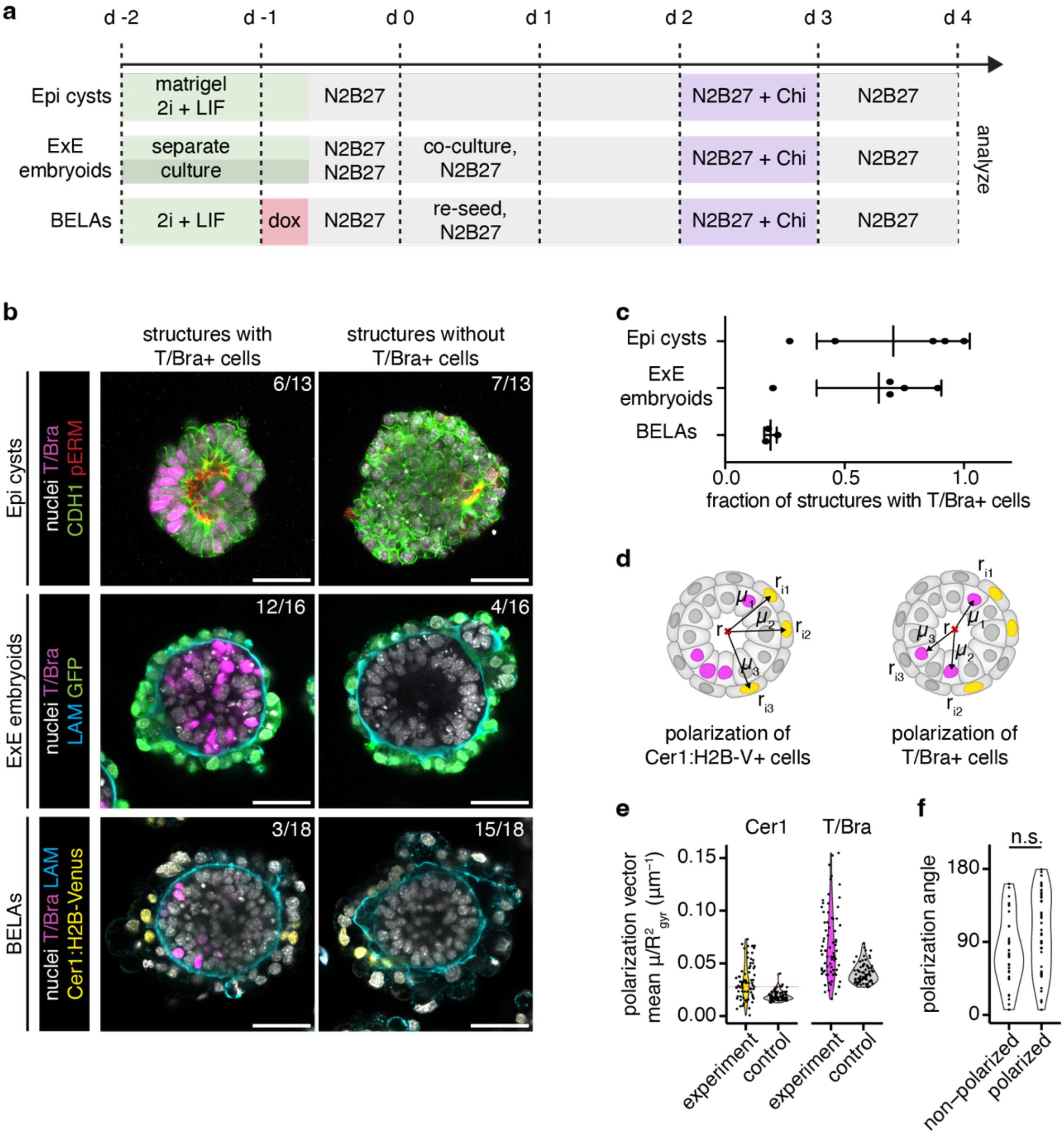

Supplementary Fig. 6. Reduced mesoderm differentiation in BELAs, related to Fig. 3.

a Schematic of the experimental approach to trigger mesoderm differentiation in Epi cysts, EXE embryoids, and BELAs. **b** Immunostaining for T/Bra (magenta) in Epi cysts (top), EXE embryoids (middle) and BELAs (bottom). Counterstains are for CDH1 (green) and pERM (red) in Epi cysts, GFP/Venus (green) to detect IM8A1 XEN cells and LAM (blue) in EXE embryoids, and GFP/Venus (yellow) to detect Cer1:H2B-Venus+ cells, and LAM in BELAs. Scale bar: 50 µm. **c** Fraction of Epi cysts, EXE embryoids and BELAs showing T/Bra-expressing cells after differentiation as in (**a**). **d** Illustration of approach to determine the polarization of Cer1:H2B-Venus+ and T/Bra+ domains in BELAs. **e** Normalized polarization vectors for Cer1:H2B-Venus expression domains (yellow), T/Bra expression domains (magenta) compared to shuffled control data (gray). Data from 38 BELAs from n = 2 independent experiments and 2 Z-slices per BELA. **f** Distribution of angles between polarization vectors of the Cer1:H2B-Venus and the T/Bra expression domains in AVE-polarized (left) and non-polarized (right) BELAs. n. s. indicates p = 0.16 (Kolmogorov-Smirnov test). Source data for (**c**, **e**) are provided in the Source Data file.

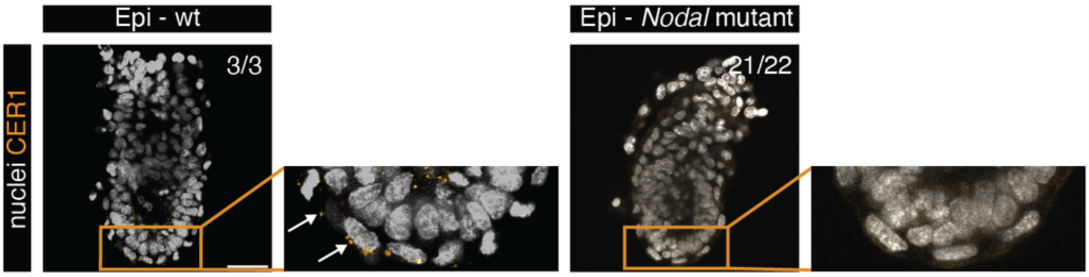

Supplementary Fig. 7. Nodal-dependent AVE differentiation in mouse embryos, related to Fig. 4. Immunostaining for CER1 (orange) in E5.5 mouse embryos generated via tetraploid complementation with wild-type (left) or *Nodal*-mutant cells (right). n = 3 (Epi – wt) and n = 22 (Epi – *Nodal* mutant) embryos, respectively.

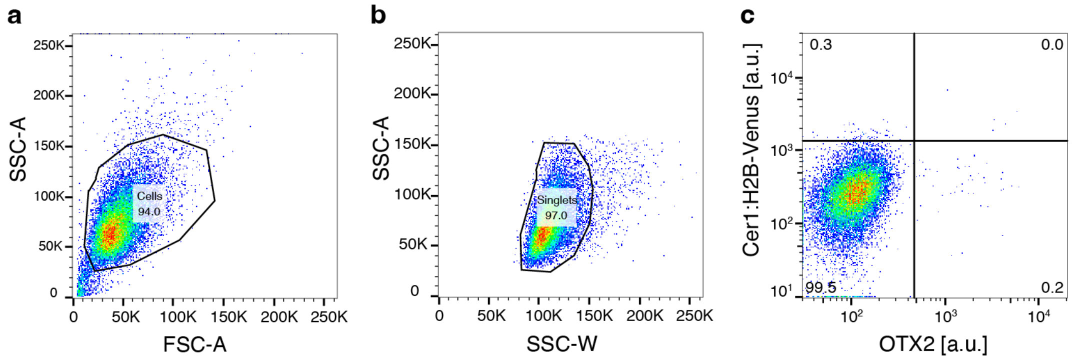

Supplementary Fig. 8. Gating strategy to identify live single cells in flow cytometry experiments, related to Fig. 4.

a Forward and side scatter of cells differentiated as shown in Fig. 4e in the absence of ActivinA and stained for OTX2 and Cer1:H2B-Venus. Black polygon shows gate used to select cells for further analysis. **b** Same as in (**a**), but showing width of side scatter. **c** OTX2 and Cer1:H2B expression of same cells as shown in (**a**, **b**), reproduced from Fig. 4g.

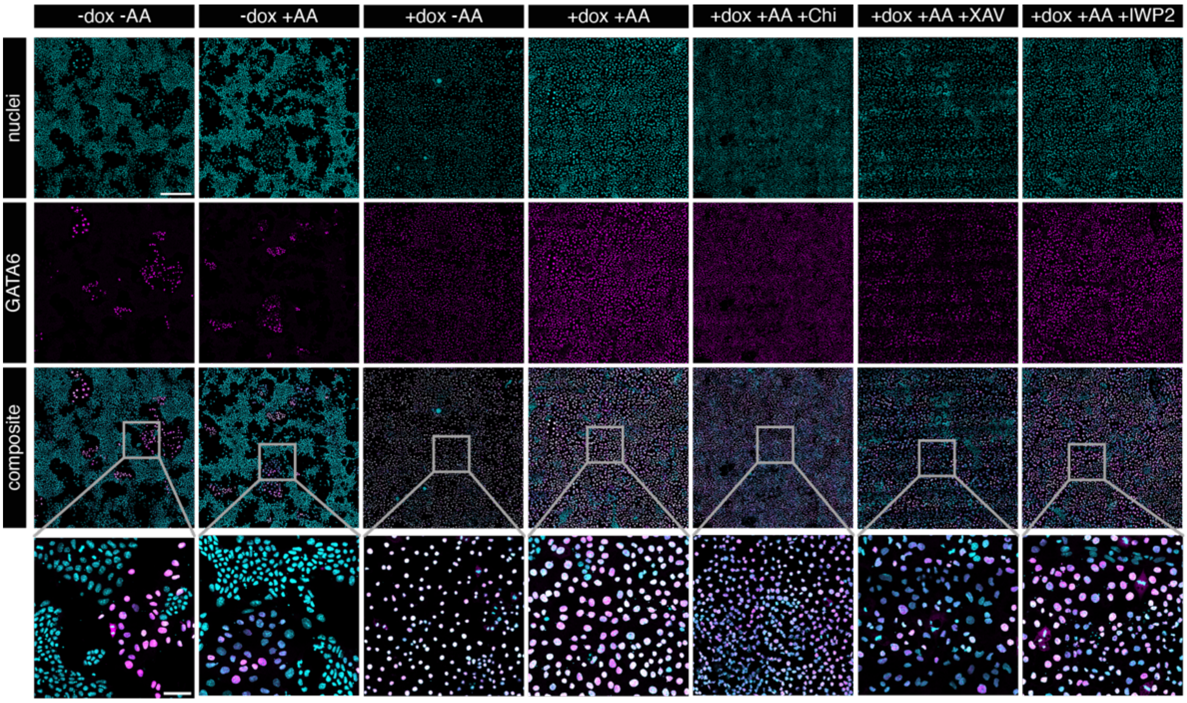

Supplementary Fig. 9. Expression of the endoderm marker GATA6 is dependent on doxycycline treatment of inducible cells, but independent from ActivinA and Wnt/β-catenin signaling, related to Figures 4 and 5.

Immunostaining of GATA4-inducible cells cultured for 3 days in the indicated media conditions without (first two columns) or with prior doxycycline induction. GATA6 expression in magenta, nuclei stained with Hoechst33342 shown in cyan. ActivinA was used at a concentration of 50 ng/ml, concentrations of all other supplements were the same as in Fig. 5. Note that a similar, low number of GATA6-positive cells is obtained in the absence of doxycycline induction both with and without ActivinA treatment, suggesting that these are a consequence of leaky transgene expression. n = 1 independent experiment. Scale bars: 250 μm (upper panels); 50 μm (bottom panels).

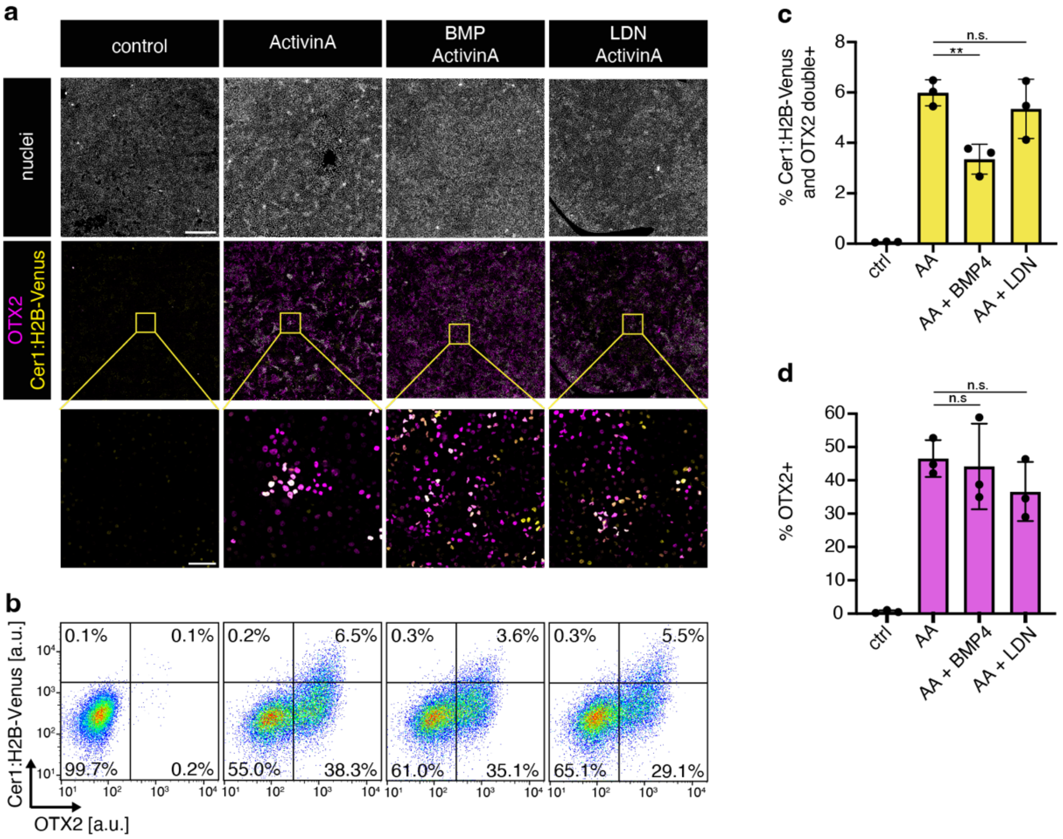

Supplementary Fig. 10. Influence of BMP signaling manipulation on AVE differentiation, related to Fig. 5.

a Immunostaining for OTX2 (magenta) and H2B-Venus (yellow) of Cer1:H2B-Venus reporter cells differentiated for 3 days after an extended doxycycline pulse with 50 ng/ml ActivinA (AA), together with 50 ng/ml BMP4 or 100 nM LDN193189 as indicated. One representative out of n = 2 independent experiments shown. **b** Flow cytometry of cells differentiated and stained as in (**a**). **c** Mean percentage of Cer1:H2B-Venus; OTX2 double-positive cells differentiated as in (**b**). n = 3 independent experiments, error bars indicate SD. ** indicates p = 0.0044 for AA vs. AA + BMP4, n. s. indicates p = 0.44 for AA vs. AA + LDN (two-tailed, unpaired t-test). **d** Same as (**c**) but showing percentage of OTX2-positive cells. n. s. indicates p = 0.78 for AA vs. AA + BMP4, and p = 0.18 for AA vs. AA + LDN (two-tailed, unpaired t-test). Data for conditions without ActivinA, and with ActivinA but without BMP signaling manipulation are the same as in Fig. 5. Scale bars: 500 µm ((**a**) overview); 50 µm ((**a**) inset). Source data for (**c**, **d**) are provided in the Source Data file.

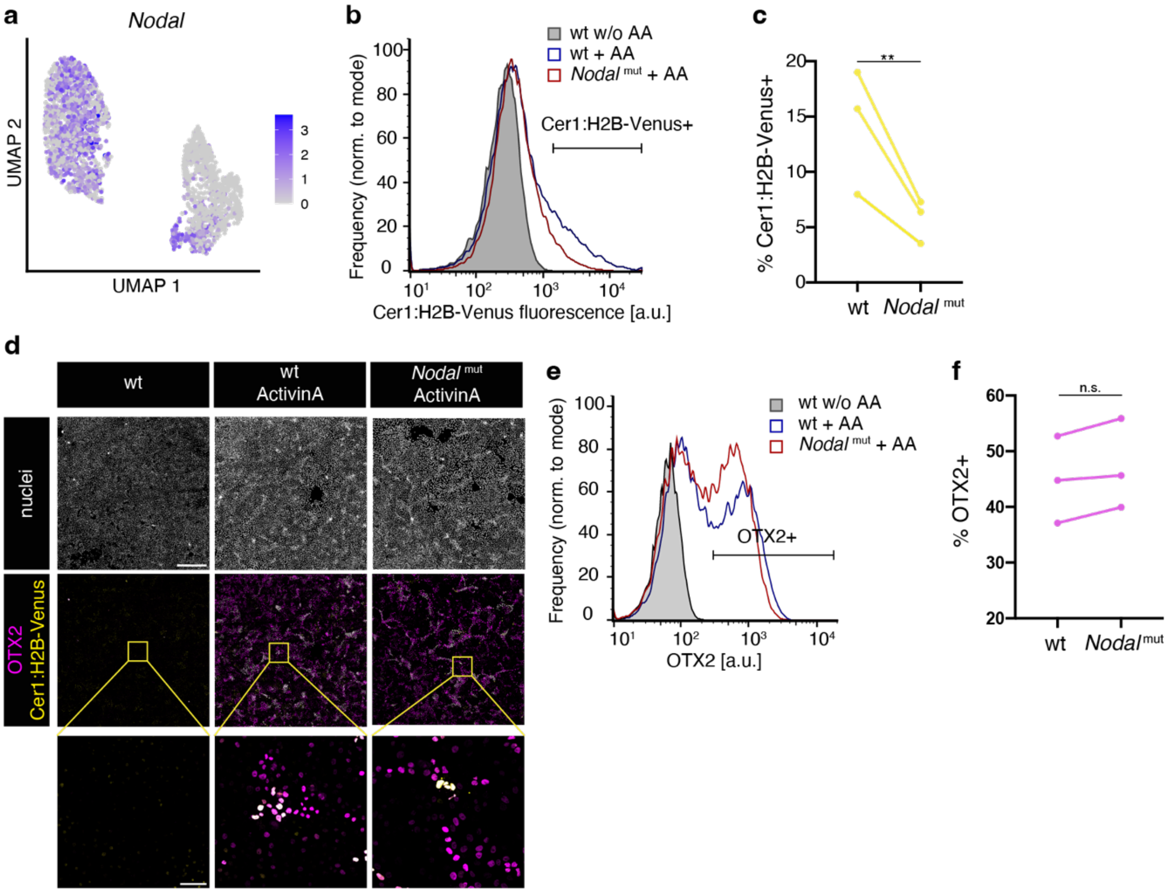

Supplementary Fig. 11. AVE differentiation in *Nodal*-mutant cells, related to Fig. 5.

a *Nodal* expression from single-cell sequencing data, shown on UMAP plot from Fig. 2b. **b** Flow cytometry of wild-type and *Nodal*-mutant Cer1:H2B-Venus reporter cells differentiated for 3 days after an extended doxycycline pulse with 50 ng/ml ActivinA. **c** Percentage of Cer1:H2B-Venus-positive cells differentiated as in (**b**) from n = 3 independent experiments. ** indicates p = 0.0021 as determined by a two-tailed, paired ratio t-test. **d** Immunostaining for OTX2 (magenta) and H2B-Venus (yellow) of wild-type and Nodal-mutant Cer1:H2B-Venus reporter cells as in (**b**). **e** Flow cytometry of OTX2 staining of cells differentiated as in (**b**). **f** Mean percentage of OTX2-positive cells differentiated as in (**b**) from n = 3 independent experiments. n.s. indicates p = 0.089 as determined by a two-tailed, paired ratio t-test. Variability in background staining intensities precluded comparison of Cer1:H2B-Venus signal in fixed and stained cells in (**d**-**f**). Data from wild-type cells with and without ActivinA are the same as in Fig. 5. Scale bars: 500 µm ((**d**) overview); 50 µm ((**d**) inset). Source data for (**c**, **f**) are provided in the Source Data file.

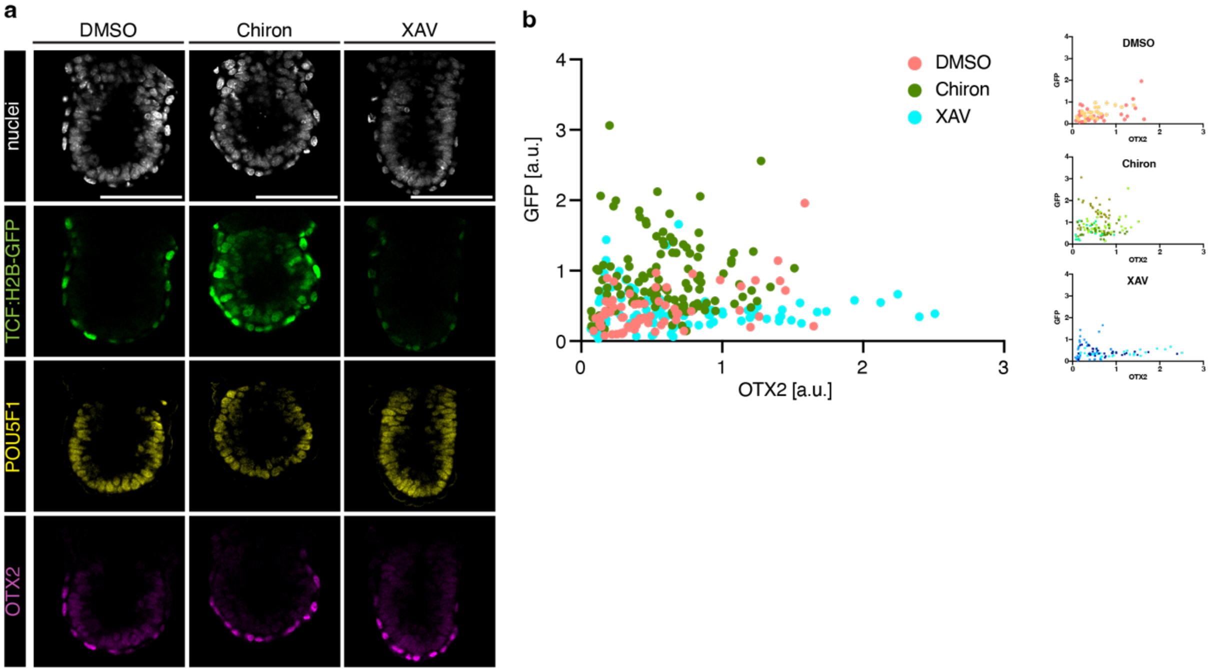

Supplementary Fig. 12. Modulation of β-catenin signaling in mouse embryos, related to Fig. 5. **a** Immunostaining for GFP (green), POU5F1 (yellow) and OTX2 (magenta) of TCF/LEF-reporter mouse embryos isolated at E5.25 followed by 24 h of culture in the presence of DMSO vehicle (left), 3 μM Chi99021 (middle) or 20 μM XAV993 (right). **b** Single cell analysis of GFP and OTX2 staining intensity in the VE of control embryos (red), Chi-treated embryos (green) and XAV-treated embryos (blue). Small panels on the right show same data color-coded for individual embryos. n ≥ 2 embryos per condition, n ≥ 66 cells in each condition. Scale bars: 100 μm. Source data for (**b**) are provided in the Source Data file.

Supplementary Reference

1. Mesnard, D., Filipe, M., Belo, J. A. & Zernicka-Goetz, M. The anterior-posterior axis emerges respecting the morphology of the mouse embryo that changes and aligns with the uterus before gastrulation. *Current biology* **14**, 184–196 (2004).
