# Supplementary material for: Tissue-intrinsic beta-catenin signals antagonize Nodal-driven anterior visceral endoderm differentiation
Source: Nat Commun. 2024 Jun 13;15:5055. doi: 10.1038/s41467-024-49380-0 (PMC11176336; doi:10.1038/s41467-024-49380-0)
Supplement: Supplementary file 3 — Description of Additional Supplementary Files [file 41467_2024_49380_MOESM3_ESM.pdf]

Description of Additional Supplementary Files

Supplementary Movie Legends:

Supplementary Movie 1. Time-lapse imaging of BELA formation, related to Fig. 1. Mixtures Epi and PrE cells were differentiated from a single culture and re-seeded in N2B27 medium on gelatin-coated dishes 16 h after the end of the doxycycline pulse. Scale bar: 200 µm, frame rate: 2 images/h.

Supplementary Movie 2. Light sheet imaging of BELA stained for GATA6 and POU5F1, related to Fig. 1. 3D rendering and animation of a BELA stained for POU5F1 (green) and GATA6 (magenta) and imaged by light sheet microscopy.

Supplementary Movie 3. Organization of basement membrane in a BELA detected by light sheet imaging, related to Fig. 1. Animation of Z-stack of same BELA as in Video S2, but now also showing Laminin staining in yellow.

Supplementary Movie 4. Light sheet imaging of Cer1:H2B-Venus expression in a BELA, related to Fig. 3. 3D rendering and animation of a BELA made from Cer1:H2B-Venus reporter cells stained for POU5F1 (green) and Cer1:H2B-Venus (yellow), and imaged by light sheet microscopy.

Supplementary Data Legends:

Supplementary Data 1. List of differentially expressed genes between clusters 3 and 4 identified in Fig. 3. Table lists differentially expressed genes (column A), p-value from Wilcoxon rank sum test (column B), and mean log2-transformed expression fold change (column C). Columns D and E list the fraction of cells where the gene was detected. In sheet "upreg_in_AVE", column D indicates fraction of cells from cluster 4 and column E indicates fraction of cells from cluster 3. In sheet "upreg_in_VE", column D indicates fraction of cells from cluster 3 and column E indicates fraction of cells from cluster 4. Column F indicates p-value after Bonferroni adjustment.

Supplementary Data 2. Output of LIANA analysis, related to Fig. 4. Table lists potential ligand-receptor interactions between Epi cells (clusters 1 and 2 in Fig. 3A), and VE cells (clusters 3 and 4 in Fig. 3A) from the BELA sample.
