# Supplementary material for: Tissue-intrinsic beta-catenin signals antagonize Nodal-driven anterior visceral endoderm differentiation
Source: Nat Commun. 2024 Jun 13;15:5055. doi: 10.1038/s41467-024-49380-0 (PMC11176336; doi:10.1038/s41467-024-49380-0)
Supplement: Supplementary file 10 — Reporting Summary [file 41467_2024_49380_MOESM10_ESM.pdf]

nature portfolio

Corresponding author(s): Christian Schröter

Last updated by author(s): Mar 18, 2024

Reporting Summary

Nature Portfolio wishes to improve the reproducibility of the work that we publish. This form provides structure for consistency and transparency in reporting. For further information on Nature Portfolio policies, see our Editorial Policies and the Editorial Policy Checklist.

Statistics

For all statistical analyses, confirm that the following items are present in the figure legend, table legend, main text, or Methods section.

n/a	Confirmed	
☐	☒	The exact sample size (*n*) for each experimental group/condition, given as a discrete number and unit of measurement
☐	☒	A statement on whether measurements were taken from distinct samples or whether the same sample was measured repeatedly
☐	☒	The statistical test(s) used AND whether they are one- or two-sided<br>*Only common tests should be described solely by name; describe more complex techniques in the Methods section.*
☒	☐	A description of all covariates tested
☐	☒	A description of any assumptions or corrections, such as tests of normality and adjustment for multiple comparisons
☐	☒	A full description of the statistical parameters including central tendency (e.g. means) or other basic estimates (e.g. regression coefficient) AND variation (e.g. standard deviation) or associated estimates of uncertainty (e.g. confidence intervals)
☐	☒	For null hypothesis testing, the test statistic (e.g. *F*, *t*, *r*) with confidence intervals, effect sizes, degrees of freedom and *P* value noted<br>*Give P values as exact values whenever suitable.*
☒	☐	For Bayesian analysis, information on the choice of priors and Markov chain Monte Carlo settings
☒	☐	For hierarchical and complex designs, identification of the appropriate level for tests and full reporting of outcomes
☐	☒	Estimates of effect sizes (e.g. Cohen's *d*, Pearson's *r*), indicating how they were calculated

Our web collection on statistics for biologists contains articles on many of the points above.

Software and code

Policy information about availability of computer code

Data collection

Leica applications suite X
MicroManager
FACS Software: DIVA
FlowJo v10.7.2
FIJI
Image Stitching FIJI plugin
Imaris 9.3.1
Illustrator

Data analysis

Cell Ranger version 4.0.0
R version 4.0.4
RStudio version 2022.12.0+353
R package Seurat version 4.1.1
R package SeuratDisk version 4.1.3
R package ggplot2 version 3.4.0
R package pheatmap version 1.0.12
R package data.table version 1.14.6
R package igraph version 1.3.5
R package tidyverse version 1.3.2
R package magrittr 2.0.3

April 2023

R package liana version 0.1.3
R package OmnipathR version 2.0.0
Python version 3.8.8
Python package scanpy version 1.8.2
Python package pandas version 1.2.4
Python package seaborn version 0.11.1
All code used for analysis and visualization, together with a list of the R packages used, is available on GitHub at https://github.com/Schroeterlab/BELAs_Schumacher_et_al.

For manuscripts utilizing custom algorithms or software that are central to the research but not yet described in published literature, software must be made available to editors and reviewers. We strongly encourage code deposition in a community repository (e.g. GitHub). See the Nature Portfolio guidelines for submitting code & software for further information.

Data

Policy information about availability of data

All manuscripts must include a data availability statement. This statement should provide the following information, where applicable:

- Accession codes, unique identifiers, or web links for publicly available datasets
- A description of any restrictions on data availability
- For clinical datasets or third party data, please ensure that the statement adheres to our policy

Single-cell RNA-sequencing data generated in this study has been deposited at the NCBI gene expression omnibus repository under accession number GSE198780. [...]. Any additional information required to reanalyze the data reported in this paper is available from the authors upon request.

Research involving human participants, their data, or biological material

Policy information about studies with human participants or human data. See also policy information about sex, gender (identity/presentation), and sexual orientation and race, ethnicity and racism.

Reporting on sex and gender: n.a.

Reporting on race, ethnicity, or other socially relevant groupings: n.a.

Population characteristics: n.a.

Recruitment: n.a.

Ethics oversight: n.a.

Note that full information on the approval of the study protocol must also be provided in the manuscript.

Field-specific reporting

Please select the one below that is the best fit for your research. If you are not sure, read the appropriate sections before making your selection.

☒ Life sciences ☐ Behavioural & social sciences ☐ Ecological, evolutionary & environmental sciences

For a reference copy of the document with all sections, see nature.com/documents/nr-reporting-summary-flat.pdf

Life sciences study design

All studies must disclose on these points even when the disclosure is negative.

Sample size: BELAs were formed by seeding iGATA cells on 6-well dishes, resulting in the spontaneous formation of hundreds of structures in each condition. For staining experiments, hundreds of structures were stained in suspension simultaneously, and at least ten structures were imaged at high resolution. Representative samples were selected for display in the figures.
For scRNAseq analysis, > 100 representative BELAs and VE cysts were manually selected and pooled for analysis.
For 2D AVE differentiation, large fields of view of ≥1mm2 were imaged, and representative areas chosen for display. Flow cytometry measurements were carried out on at least 20.000 cells per experiment and condition. Sample size for embryo experiments was determined by litter sizes.

Data exclusions: No data were excluded from the analysis.

Replication: Experiments involving BELA formation and staining as well as 2D AVE differentiation were repeated at least 3 times. The scRNAseq experiment reported in this study was performed only once, but critical results were subsequently confirmed using independent experimental approaches, such as staining for markers.

Randomization: n.a.

Blinding | n.a.

Reporting for specific materials, systems and methods

We require information from authors about some types of materials, experimental systems and methods used in many studies. Here, indicate whether each material, system or method listed is relevant to your study. If you are not sure if a list item applies to your research, read the appropriate section before selecting a response.

Materials & experimental systems

n/a	Involved in the study
☐	☒ Antibodies
☐	☒ Eukaryotic cell lines
☒	☐ Palaeontology and archaeology
☐	☒ Animals and other organisms
☒	☐ Clinical data
☒	☐ Dual use research of concern
☒	☐ Plants

Methods

n/a	Involved in the study
☒	☐ ChIP-seq
☐	☒ Flow cytometry
☒	☐ MRI-based neuroimaging

Antibodies

Antibodies used

Mouse anti-Oct3/4 Santa Cruz Biotechnology Cat# sc-5279; RRID: AB_628051; dilution 1:100 - 1:200
Goat anti-GATA6 R&D Systems Cat# AF1700; RRID: AB_2108901; dilution 1:200
Goat anti-SOX17; R&D Systems Cat# AF1924; RRID: AB_355060; dilution 1:200
Rat anti-PODXL; R&D Systems Cat# MAB1556; RRID: AB_2166010; dilution 1:200
Rabbit anti-pERM; Cell Signaling Technology Cat# 3726; RRID: AB_10560513; dilution 1:200
Alexa Fluor 647 Hamster anti-Rat CD29; BD Biosciences Cat# 562153; RRID: AB_10896298; dilution 1:100
Rat anti-E-Cadherin; Takara Bio Cat# M108; RRID: AB_2895157; dilution 1:200
Rabbit anti-ZO-1; Thermo Fisher Scintific Cat# 61-7300; RRID: AB_2533938; dilution 1:200
Chicken anti-GFP; Abcam Cat# ab13970; RRID: AB_300798; dilution 1:200
Goat anti-OTX2; Neuromics Cat# GT15095; RRID: AB_2157174; dilution 1:200
Rabbit anti-LAM; Sigma Aldrich Cat# L9393; RRID: AB_477163; dilution 1:500 - 1:750
Rat anti-CER1; R&D Systems Cat# MAB1986; RRID: AB_2275974; dilution 1:200
Mouse anti-Oct4; Cell signaling technology Cat# 83932, RRID:AB_2721046; dilution 1:200
Goat anti-Otx2; R&D systems Cat# AF1979; RRID: AB_2157172; dilution 1:200
Rabbit anti-Oct-4A (D6C8T); Cell signalling Cat# 83932S; RRID: AB_2721046; dilution 1:200

Validation

Binding specificity of antibodies was assessed within the study through the specific staining of subsets of cells or extracellular structures in BELAs or 2D differentiation protocols.

Eukaryotic cell lines

Policy information about cell lines and Sex and Gender in Research

Cell line source(s)

All embryonic stem cell lines used in this study were on an E14tg2a background (Hooper et al., 1987).
The inducible Tet::GATA4-mCherry (iGATA) lines have been generated by our group and were previously described (Raina et al., 2021).
The XEN cell lines IM8A1-GFP (Kunath et al., 2007) and X10 (Brown et al., 2010) have previously been described and where kindly shared by Kat Hadjantonakis.
Cer1:H2B-Venus and Nodal-mutant iGATA cell lines were generated in this study.

Authentication

Cell lines were validated by their expression of ESC- and XEN-specific markers, as well as their ability to contribute to BELA development

Mycoplasma contamination

All cell lines were regularly tested negative for mycoplasma contamination.

Commonly misidentified lines (See ICLAC register)

n.a.

Animals and other research organisms

Policy information about studies involving animals; ARRIVE guidelines recommended for reporting animal research, and Sex and Gender in Research

Laboratory animals

Mouse (Mus musculus): WT B6C3F1 or CD1 strains bred in house
Mouse (Mus musculus): TCF/Lef:H2B-GFP described in Ferrer-Vaquer A., et al., BMC developmental biology 10, 121 (2010); JAX: 013752, C57BL/6-TCF/Lef1-HIST1H2BB/EGFP

	The mice used in this study were at age from 6 weeks to 5 months. Animals were maintained under a 14-hour light/10-hour dark cycle with free access to food and water, at 21.5ºC ambient temperature and 55% - 65% humidity.
Wild animals	n.a.
Reporting on sex	sex of embryos was not determined in this study
Field-collected samples	n.a.
Ethics oversight	Animal expeirments and husbandry were performed according to the German Animal Welfare guidelines and approved by the Landesamt für Natur, Umwelt und Verbraucherschutz Nordrhein-Westfalen (State Agency for Nature, Environment and Consumer Protection North-Rhine-Westphalia)

Note that full information on the approval of the study protocol must also be provided in the manuscript.

Plants

Seed stocks	n.a.
Novel plant genotypes	n.a.
Authentication	n.a.

Flow Cytometry

Plots

Confirm that:

☒ The axis labels state the marker and fluorochrome used (e.g. CD4-FITC).

☒ The axis scales are clearly visible. Include numbers along axes only for bottom left plot of group (a 'group' is an analysis of identical markers).

☒ All plots are contour plots with outliers or pseudocolor plots.

☒ A numerical value for number of cells or percentage (with statistics) is provided.

Methodology

Sample preparation	Cells for flow cytometry were detached from culture vessels and either analyzed immediately, or fixed in 4% paraformaldehyde for 15 min, washed with PBS and then incubated in PBS + 1% BSA + 0.25% Saponin (PBSap) for 30 min at room temperature. Afterwards, cells were incubated with primary antibodies diluted in PBSap at 4ºC overnight. The next day, cells were washed three times in PBSap and incubated with secondary antibodies diluted in PBSap for at least one hour. Cells were washed three times in PBSap, and passed through a cell strainer.
Instrument	Fixed and stained cells were analyzed on a LSRII flow cytometer (BD Biosciences). Live cells were sorted on a FACS Aria Fusion (BD Biosciences).
Software	Data were collected with FACS Diva, and analyzed with FlowJo (both from BD Biosciences).
Cell population abundance	Abundances of relevant populations differed from experiment to experiment and are given in the relevant figure items.
Gating strategy	Single, viable cells were identified and gated for analysis based on the area as well as the width of their forward and side scatter properties.

☒ Tick this box to confirm that a figure exemplifying the gating strategy is provided in the Supplementary Information.
